# Supplementary material for: Endemic-epidemic modelling of school closure to prevent spread of COVID-19 in Switzerland
Source: BMC Infect Dis. 2024 Dec 2;24:1372. doi: 10.1186/s12879-024-09674-6 (PMC11610105; doi:10.1186/s12879-024-09674-6)
Supplement: Supplementary file 1 — Supplementary Material 1 [file 12879_2024_9674_MOESM1_ESM.pdf]

Supporting information for “Endemic-epidemic modelling for school closure to prevent spread of COVID-19 in Switzerland”

This document contains ancillary information which is relevant to the manuscript. It consists of additional figures, an outline of the model selection procedure used, and a sensitivity analysis of time-varying transmission weights used in the modelling approach.

1 Additional figures

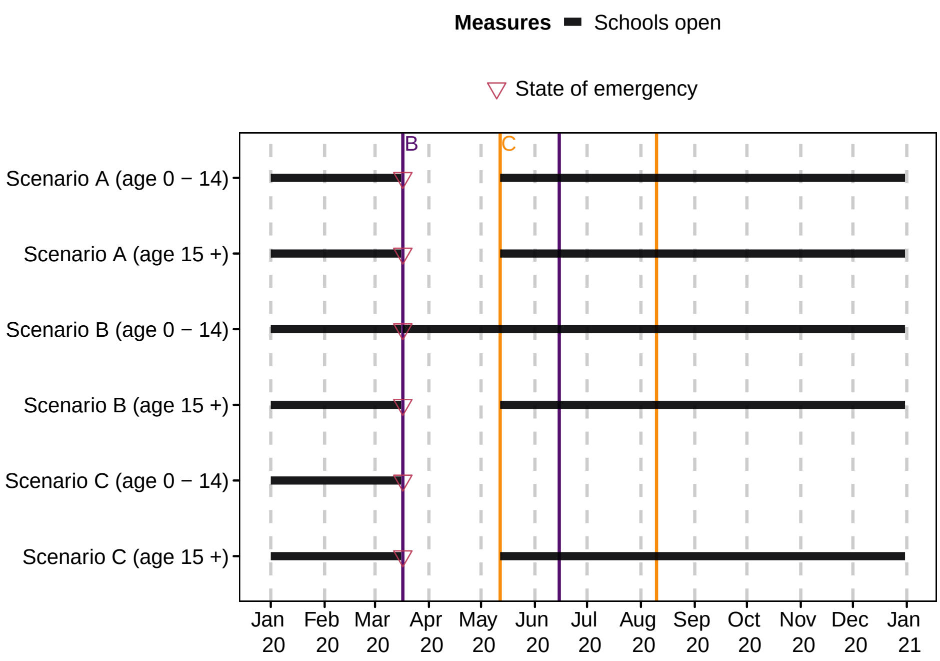

Figure S1: Illustration of scenarios. The state of emergency in Switzerland leading to “lockdown” was declared on 17th March 2020 (vertical line between March and April 2020). The projection windows are shown with coloured vertical lines

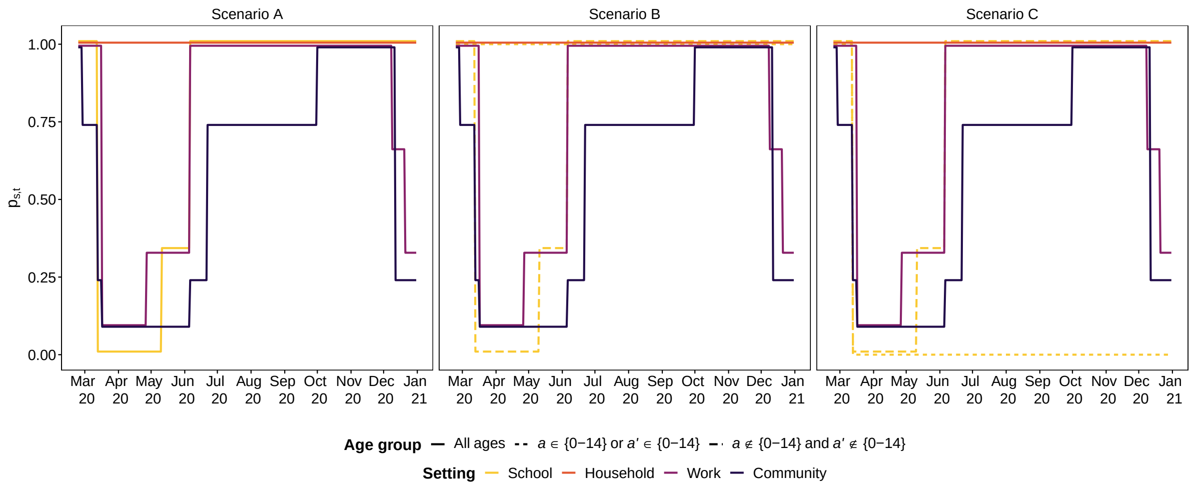

Figure S2: Policy information $p_{s,t,a,a'}$ for the scenarios considered. A slight jitter has been applied to assist in the visualisation

2 Model selection procedure

The reference model is

$$Y_{at} \mid Y_{a,t-1}, \ldots, Y_{a,t-d_{\text{max}}} \sim \text{NegBin}(\lambda_{at}, \psi)$$

$$\lambda_{at} = \underbrace{\nu_{at} e_a}_{\text{endemic}} + \underbrace{\phi_{at} \sum_{a'} \sum_{d=1}^{d_{\text{max}}} u_d w_{a,a',t} Y_{a',t-d}}_{\text{epidemic}} \tag{1}$$

$$\log(\nu_{at}) = \alpha_a^{(\nu)} + \beta_{\text{public holiday}}^{(\nu)} x_t \tag{2}$$

$$\log(\phi_{at}) = \alpha_a^{(\phi)} + \beta_{\text{public holiday}}^{(\phi)} x_t \tag{3}$$

$$u_d \propto \frac{\kappa^{d-1}}{(d-1)!} \cdot \exp(-\kappa), \quad \kappa > 0, \quad d = 1, \ldots, d_{\text{max}}$$

This is denoted model 0 in our statistical analysis plan (can be found in our study protocol at https://osf.io/fgrdy). The reference model has fixed effects of age group, public holiday score, and school holiday score in both components. The model fit is shown in Figure S4. Overall, the predicted values (shaded area) seem to fit the observed data (points) quite well. During the first wave (here we consider cases before 1st June 2020 to be the first oscillation in the absence of a true definition of epidemic waves), the model overestimates the number of cases for all age groups except the youngest age group. This is seen in the shaded area being greater than the observed counts. We can also see that there might be some

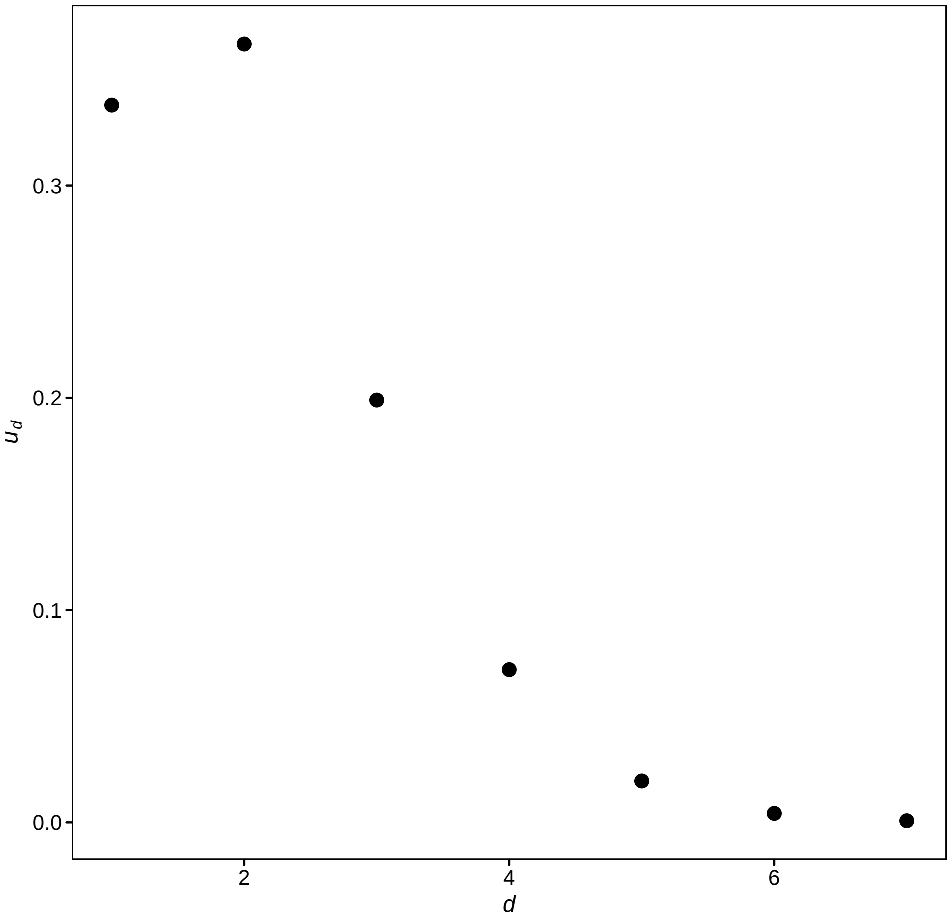

Figure S3: Estimated lag distribution $\hat{u_d}$ (below)

uncaptured weekday effects as the observed cases seem to oscillate more within a week than is captured by the model. According to the reference model, nearly all cases can be captured by the epidemic component ϕ.

The model parameter estimates for the reference model are given in Table S1. Here we also experienced having a large standard error for the fixed effect of the oldest age group in the endemic component $\alpha_{80+}^{(\nu)}$. Dropping this coefficient from the model resolved the issue. This model has smaller values for $\alpha^{(\nu)}$ than the final model selected (estimates are shown in the main manuscript). The public holiday estimates are greater in this model. This implies some of the effect they capture here is explained by other effects when they are included in the model, such as those capturing the weekly fluctuations in cases. Overdispersion ψ is also much larger than in the final model and the decreasing pattern found for $\alpha^{(\phi)}$ is not evid-

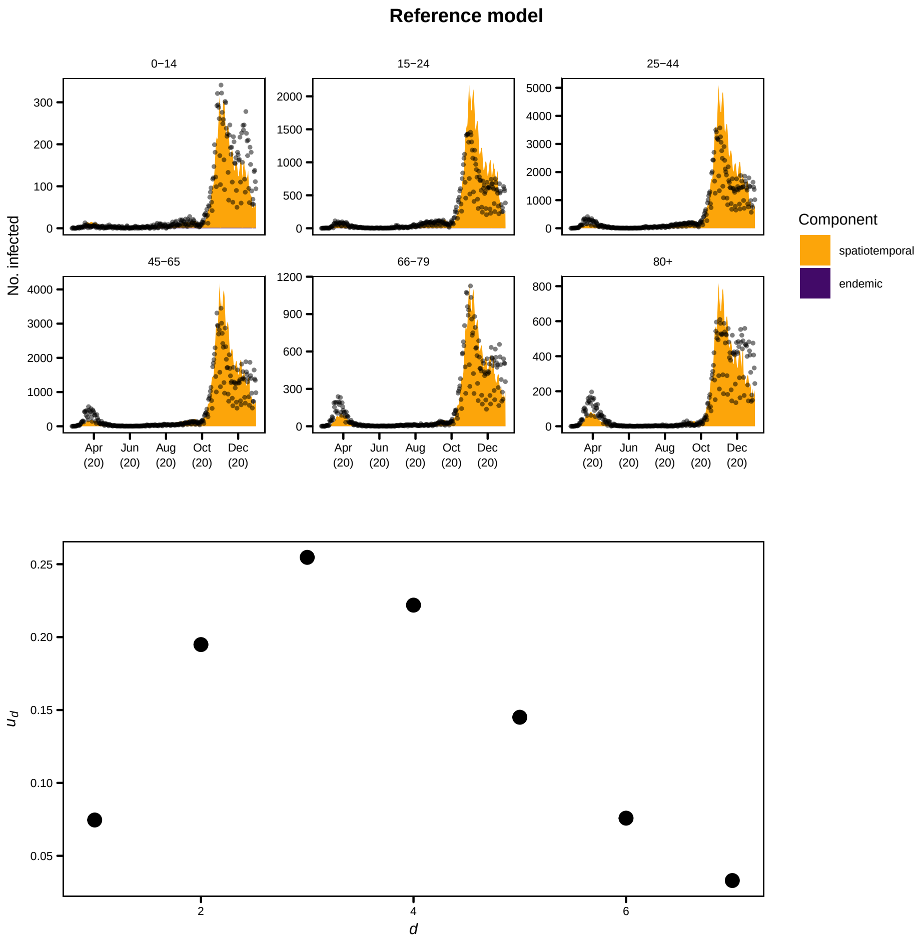

Figure S4: Reference model with age group specific effects as well as public holiday score in both components

ent for the reference model.

When constructing models in addition to the effects considered in the reference model, we considered covariates for sine-cosine waves (non-linear trend), temperature, testing rate, time trend (linear trend), weekday, and weekend. As certain covariates capture similar effects we did not fit models for all possible predictor combinations. Mutually exclusive effects within the same model component are weekday and weekend as well as temperature and non-linear trend represented through sine-cosine waves. The remaining covariates do not have any

Table S1: Reference model coefficients

Endemic – $\log(\nu_{at})$			Epidemic – $\log(\phi_{at})$			Other parameters		
Coefficient	Estimate	Std. Error	Coefficient	Estimate	Std. Error	Coefficient	Estimate	Std. Error
$\alpha^{(\nu)}_{0\text{-}14}$	2.002	0.178	$\alpha^{(\phi)}_{0\text{-}14}$	-3.607	0.045	ψ_{0-14}	0.278	0.032
$\alpha^{(\nu)}_{15\text{-}24}$	2.910	0.309	$\alpha^{(\phi)}_{15\text{-}24}$	-2.131	0.044	ψ_{15-24}	0.318	0.028
$\alpha^{(\nu)}_{25\text{-}44}$	1.907	0.522	$\alpha^{(\phi)}_{25\text{-}44}$	-1.983	0.040	ψ_{25-44}	0.315	0.026
$\alpha^{(\nu)}_{45\text{-}65}$	0.805	1.142	$\alpha^{(\phi)}_{45\text{-}65}$	-2.066	0.043	ψ_{45-65}	0.389	0.032
$\alpha^{(\nu)}_{66\text{-}79}$	1.273	0.627	$\alpha^{(\phi)}_{66\text{-}79}$	-1.802	0.043	ψ_{66-79}	0.382	0.035
			$\alpha^{(\phi)}_{80+}$	-0.896	0.048	ψ_{80+}	0.583	0.056
$\beta^{(\nu)}_{\text{public holiday}}$	-1.944	1.396	$\beta^{(\phi)}_{\text{public holiday}}$	-0.407	0.110			
						$\log\kappa$	0.961	

restrictions. Bayesian information criterion was used to determine goodness-of-fit of the models considered in this work. Table S2 shows the Bayesian information criterion values for the models considered. The selected model presented in the main manuscript is the model with option 14 for $\log(\nu_{at})$ and option 13 for $\log(\phi_{at})$ as described in our statistical analysis plan, which is available at https://osf.io/fgrdy..

Table S2: Models ranked by Bayesian information criterion (BIC)

	Endemic *nu*						Epidemic *phi*						
Rank	Weekday	Weekend	Time trend	Testing rate	Temperature	Sine-cosine wave	Weekday	Weekend	Time trend	Testing rate	Temperature	Sine-cosine wave	BIC
1		✓				✓	✓					✓	17,224
2	✓					✓	✓					✓	17,139
3			✓			✓	✓					✓	17,123
4						✓	✓					✓	16,513
5			✓	✓			✓					✓	17,126
6			✓			✓	✓				✓		16,981
7			✓			✓	✓						17,032
8			✓			✓	✓			✓			16,402
9			✓				✓					✓	17,109
10		✓			✓		✓					✓	16,946
11			✓		✓		✓					✓	17,007
12					✓		✓					✓	16,499
13		✓					✓					✓	16,437
14	✓				✓		✓					✓	16,551
15							✓					✓	16,418
16	✓					✓		✓				✓	16,376
17	✓						✓					✓	16,478
18		✓		✓			✓					✓	16,495
19		✓				✓		✓				✓	16,464
20			✓			✓		✓				✓	16,012
21						✓		✓				✓	16,458
22			✓		✓			✓				✓	16,493
23		✓				✓	✓				✓		16,459
24	✓					✓	✓				✓		15,924
25		✓				✓	✓			✓			16,335
26			✓			✓		✓					16,374

Table S2: Models ranked by Bayesian information criterion (BIC) *(continued)*

	Endemic *nu*						Epidemic *phi*						
Rank	Weekday	Weekend	Time trend	Testing rate	Temperature	Sine-cosine wave	Weekday	Weekend	Time trend	Testing rate	Temperature	Sine-cosine wave	BIC
27			✓			✓		✓			✓		16,341
28			✓			✓		✓		✓			16,012
29						✓	✓				✓		15,871
30	✓					✓	✓			✓			15,852
31		✓				✓	✓						15,821
32			✓	✓			✓				✓		15,590
33	✓					✓	✓						16,591
34						✓	✓			✓			16,599
35						✓	✓						16,582
36			✓	✓			✓			✓			16,121
37			✓	✓			✓						16,561
38		✓			✓			✓				✓	16,577
39					✓			✓				✓	16,566
40		✓						✓				✓	16,035
41								✓				✓	16,451
42			✓				✓				✓		16,465
43		✓				✓		✓		✓			16,456
44			✓		✓		✓				✓		16,115
45	✓				✓			✓				✓	16,010
46	✓							✓				✓	16,008
47	✓					✓		✓		✓			15,988
48			✓	✓				✓			✓		15,804
49		✓				✓		✓					17,033
50						✓		✓		✓			17,062
51	✓					✓		✓					17,028
52						✓		✓					16,818
53			✓				✓						17,037
54			✓		✓		✓						16,715
55	✓					✓		✓			✓		17,045
56		✓				✓		✓			✓		16,933
57			✓	✓				✓					16,928
58						✓		✓			✓		16,875
59			✓					✓			✓		16,897
60			✓		✓			✓					16,824
61			✓					✓					16,496
62		✓				✓			✓	✓			16,336
63	✓					✓			✓			✓	16,503
64	✓					✓						✓	16,744
65	✓					✓			✓	✓			17,222
66		✓				✓			✓			✓	17,116
67	✓					✓			✓		✓		17,100
68			✓				✓			✓			17,298
69			✓		✓		✓			✓			17,252
70					✓		✓			✓			17,284
71		✓				✓						✓	17,255
72		✓			✓		✓			✓			16,351
73			✓			✓			✓			✓	17,099
74			✓		✓				✓	✓			
75	✓				✓		✓			✓			17,066
76			✓		✓			✓			✓		17,275
77		✓			✓		✓				✓		16,441
78					✓		✓						16,387
79	✓					✓			✓				16,359
80			✓			✓				✓			16,339
81		✓			✓		✓						16,446
82					✓		✓				✓		16,470
83		✓		✓			✓				✓		16,439
84				✓			✓				✓		16,263
85		✓					✓				✓		16,388
86			✓	✓						✓			16,419
87						✓			✓			✓	16,395
88		✓				✓				✓			15,907
89			✓						✓			✓	16,282
90			✓			✓			✓	✓			16,330
91	✓				✓		✓				✓		16,290
92		✓				✓			✓		✓		16,270

Table S2: Models ranked by Bayesian information criterion (BIC) *(continued)*

	Endemic *nu*						Epidemic *phi*						
Rank	Weekday	Weekend	Time trend	Testing rate	Temperature	Sine-cosine wave	Weekday	Weekend	Time trend	Testing rate	Temperature	Sine-cosine wave	BIC
93							✓				✓		15,858
94	✓				✓		✓						15,818
95			✓			✓							15,789
96	✓			✓			✓				✓		15,597
97			✓			✓					✓		16,573
98			✓	✓							✓		16,586
99			✓	✓					✓		✓		16,569
100			✓			✓						✓	16,433
101	✓					✓				✓			
102				✓			✓			✓			16,592
103			✓	✓					✓	✓			
104	✓						✓				✓		16,802
105		✓		✓			✓			✓			16,423
106						✓			✓	✓			
107			✓	✓									16,439
108		✓				✓					✓		16,919
109			✓					✓				✓	15,990
110		✓				✓							15,966
111	✓			✓			✓			✓			15,948
112					✓			✓		✓			15,809
113			✓	✓				✓				✓	16,652
114			✓					✓		✓			16,616
115						✓					✓		16,578
116			✓			✓			✓		✓		16,445
117						✓							
118		✓			✓			✓		✓			16,797
119		✓					✓			✓			16,789
120						✓				✓			16,389
121	✓			✓					✓			✓	16,650
122			✓						✓	✓			
123							✓			✓			16,574
124					✓			✓					16,301
125		✓			✓			✓					16,396
126				✓			✓						16,178
127		✓		✓			✓						16,150
128				✓				✓			✓		16,364
129		✓		✓				✓			✓		17,139
130		✓					✓						16,996
131	✓				✓			✓					16,991
132					✓			✓			✓		16,503
133	✓						✓			✓			17,222
134	✓			✓				✓			✓		16,955
135		✓						✓			✓		17,220
136	✓				✓			✓			✓		16,384
137							✓						17,127
138	✓			✓								✓	16,944
139	✓			✓					✓		✓		16,985
140			✓		✓				✓			✓	16,496
141	✓							✓			✓		16,435
142								✓			✓		16,551
143	✓			✓			✓						16,416
144	✓						✓						16,384
145			✓		✓						✓		16,371
146						✓			✓				16,392
147			✓		✓								16,349
148		✓			✓				✓			✓	15,944
149		✓				✓			✓				16,347
150			✓								✓		16,377
151	✓				✓				✓			✓	16,343
152			✓		✓							✓	15,849
153			✓										16,341
154				✓					✓			✓	16,365
155			✓									✓	16,331
156		✓		✓					✓			✓	15,951
157					✓				✓			✓	15,813
158	✓					✓					✓		15,780

Table S2: Models ranked by Bayesian information criterion (BIC) *(continued)*

	Endemic *nu*						Epidemic *phi*						
Rank	Weekday	Weekend	Time trend	Testing rate	Temperature	Sine-cosine wave	Weekday	Weekend	Time trend	Testing rate	Temperature	Sine-cosine wave	BIC
159	✓					✓							15,750
160				✓				✓					15,586
161		✓		✓				✓					16,488
162		✓						✓		✓			16,488
163								✓		✓			16,472
164		✓			✓				✓	✓			16,069
165	✓			✓				✓					16,460
166		✓							✓	✓			16,472
167		✓						✓					16,460
168		✓							✓			✓	15,978
169	✓							✓		✓			16,470
170								✓					16,474
171	✓			✓				✓		✓			16,909
172	✓							✓					16,330
173	✓								✓			✓	16,045
174		✓		✓					✓		✓		16,015
175	✓								✓	✓			16,020
176			✓		✓				✓		✓		15,805
177						✓						✓	16,834
178									✓			✓	16,853
179					✓				✓	✓			16,825
180									✓	✓			16,793
181		✓		✓								✓	16,761
182			✓	✓					✓			✓	16,483
183		✓			✓							✓	16,611
184			✓	✓								✓	16,385
185						✓			✓		✓		16,816
186	✓				✓							✓	16,801
187		✓										✓	16,760
188	✓											✓	16,625
189	✓			✓					✓				16,679
190			✓			✓			✓				16,237
191		✓			✓				✓		✓		16,365
192				✓					✓		✓		16,437
193		✓		✓					✓	✓			16,810
194			✓						✓		✓		16,703
195	✓			✓					✓	✓			16,701
196					✓							✓	16,520
197	✓				✓				✓		✓		16,935
198			✓	✓				✓		✓			16,482
199												✓	16,666
200					✓				✓		✓		16,677
201			✓						✓				16,800
202			✓		✓				✓				16,686
203		✓							✓		✓		16,674
204									✓		✓		16,509
205	✓								✓		✓		16,642
206	✓				✓				✓				16,160
207		✓			✓				✓				16,284
208		✓			✓			✓			✓		16,386
209			✓		✓			✓		✓			15,658
210					✓				✓				15,679
211			✓	✓					✓				15,647
212				✓								✓	15,615
213	✓				✓						✓		
214	✓				✓								
215	✓			✓							✓		15,684
216	✓			✓									15,530
217		✓			✓						✓		15,637
218		✓									✓		15,649
219	✓										✓		15,618
220		✓			✓								15,622
221		✓							✓				15,461
222		✓		✓									15,408
223									✓				15,389
224				✓					✓				15,440

Table S2: Models ranked by Bayesian information criterion (BIC) *(continued)*

	Endemic *nu*						Epidemic *phi*						
Rank	Weekday	Weekend	Time trend	Testing rate	Temperature	Sine-cosine wave	Weekday	Weekend	Time trend	Testing rate	Temperature	Sine-cosine wave	BIC
225		✓		✓					✓				15,944
226	✓								✓				15,962
227		✓			✓					✓			15,938
228					✓					✓			16,418
229		✓								✓			
230					✓								
231	✓									✓			
232		✓											16,432
233				✓									15,935
234					✓						✓		15,953
235	✓												15,926
236											✓		15,743
237		✓		✓							✓		15,738
238				✓							✓		15,668
239										✓			15,687
240													15,719
241				✓						✓			16,642
242		✓		✓						✓			16,607
243			✓		✓					✓			16,582
244	✓			✓						✓			16,361
245			✓							✓			16,518
246	✓				✓					✓			16,445
247				✓				✓		✓			16,523
248		✓		✓				✓		✓			16,667
249	✓				✓			✓		✓			16,533
250				✓					✓	✓			16,507
251	✓				✓				✓	✓			16,500
252				✓			✓					✓	16,486
253	✓			✓			✓					✓	16,354
254				✓				✓				✓	16,152
255	✓			✓				✓				✓	16,228
256		✓		✓				✓				✓	16,295

3 Sensitivity analysis of contact matrices – model fit

The modelling approach used is dependent on the transmission weights $w_{a,a',t}$ used to inform the model. For this reason, we elect to do a sensitivity analysis. In particular, we consider it unrealistic that school contacts decrease and other contacts would be expected to remain the same so we conducted a sensitivity analysis of the situation where other contacts change to reflect this. We examine the robustness of the time-varying contact matrix $w_{a,a',t}$ through various alternatives for its construction. The total average contacts at time t are given by the weighted sum

$$w_{a,a',t} = \sum_s \gamma_{s,t} \cdot c_{a,a',s} = \sum_s d_s \cdot p_{s,t} \cdot h_{s,t} \cdot c_{a,a',s} \tag{4}$$

where s denotes setting the contact occurred in, d_s are the disease-specific weights, $p_{s,t}$ is policy, and $h_{s,t}$ is adjustments for school holidays. The setting-specific con-

tacts $c_{a,a',s}$ are the same as the ones displayed in Figure 2 in the main manuscript. The policy indicators $p_{s,t}$ used in this sensitivity analysis are the ones shown in Figure 3 of the main manuscript. We now consider the situation where d_s are allowed to vary by time as well as setting: $d_{s,t}$. Previously we considered d_s to take the values 11.41 for school setting, 8.07 for work setting, 4.11 for household, and 2.79 for other setting for all time points t. We conduct two sensitivity analyses to reflect that reductions in school contacts are unlikely to exist in a vacuum and there is likely to be some symbiosis with work contacts. Namely, when children are home from school guardians are more likely to also be home to engage in childcare. The two options for sensitivity analysis are:

Option 1: The household weight $d_s|_{\text{household}}$ reflects the school closure policy and is given by $d_s|_{\text{household}} = 2 - p_{s,t}|_{\text{school}}$

Option 2: The household weight $d_s|_{\text{household}}$ is adjusted by any amount which reduces contacts on school holidays (the adjustment is now through $h_{s,t}$)

3.1 Option 1: Household contacts depend on school closures

The first option adjusts the household indicator such that it increases when the school indicator decreases. The rationale behind this is that when schools are closed, there are fewer school contacts but more household contacts so we considered that households should "mirror" schools to reflect expected increases in contacts due to school closures. The downside of this adjustment is that it does not take into account the school holiday score $h_{s,t}$ which means that if schools are generally open but it is a holiday in most cantons, household contacts are not increased (this will be addressed in Option 2). The implications of this adjustment on all involved components can be seen in Figure S5.

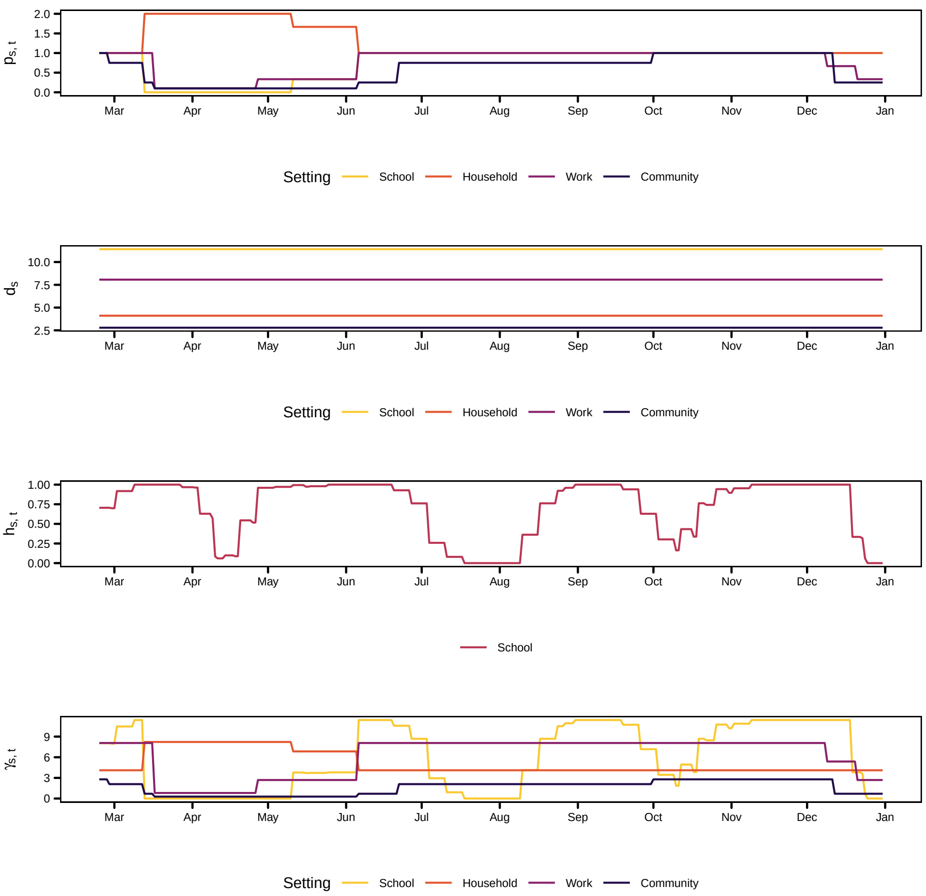

Figure S5: Time series of policy indicators $p_{s,t}$, disease weights d_s, and non-pandemic school holiday score $h_{s,t}$. The lower panel shows the product of all of the time series $\gamma_{s,t}$ which will serve as a multiplication factor for the setting specific contact matrix $c_{a,a',t}$

The fit and lag distribution of the model with contact matrices constructed under Option 1 is shown in Figure S6 and the corresponding parameters can be found in Table S3. Looking at the models fitted with the contact matrices constructed as explained under Option 1, the best model according to Bayesian information criterion has model covariates age, public holidays, weekend, and sine-cosine wave in the endemic component and age, public holidays, weekday, and sine-cosine wave in the epidemic component. The fit and lag distribution are shown in Figure S6.

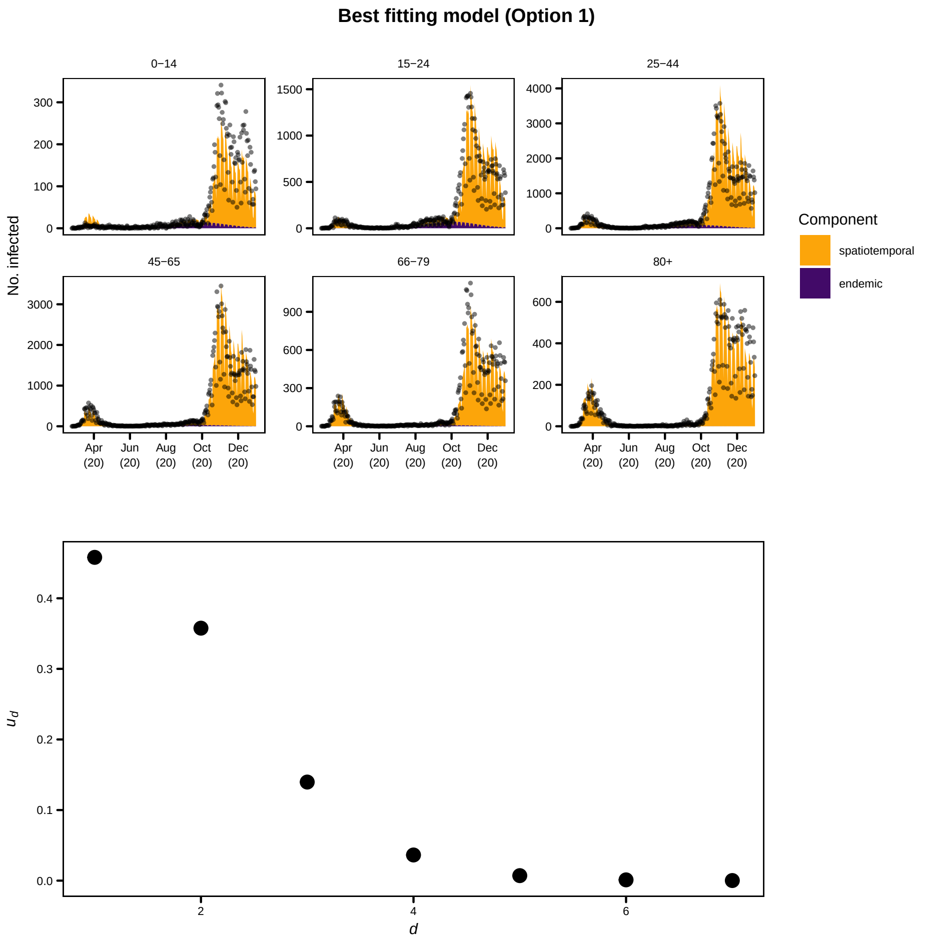

Figure S6: Model fit and lag distribution for the best model with weights as constructed under Option 1. The endemic component was fitted with effects for age, public holidays, weekend, and sine-cosine wave whereas the epidemic mean is fitted with effects for age, public holidays, weekday, and sine-cosine wave

When it comes to Option 1, we can see from the fit shown in Figure S6 that the number of cases in the youngest age group is clearly overestimated in the first wave (seen around April where the shaded area is above the points) and underestimated in the second wave (where the shaded area is below the points). The plot also shows that the contribution of the endemic mean $\hat{\nu}_{at}$ contributes predominantly in the younger age groups whereas for the older age groups almost all of the cases are attributed to the epidemic component $\hat{\phi}_{at}$. Furthermore, the estimated lag distribution $\hat{u}_d$ indicates that the maximum lag peaks at 1 which is not what we

Table S3: Coefficients of the best model using BIC under Option 1 and Scenario A.

Endemic – $\log(\nu_{at})$			Epidemic – $\log(\phi_{at})$			Other parameters		
Coefficient	Estimate	Std. Error	Coefficient	Estimate	Std. Error	Coefficient	Estimate	Std. Error
$\alpha^{(\nu)}_{\text{0-14}}$	2.617	0.195	$\alpha^{(\phi)}_{\text{0-14}}$	-3.995	0.054	ψ_{0-14}	0.256	0.032
$\alpha^{(\nu)}_{\text{15-24}}$	4.601	0.175	$\alpha^{(\phi)}_{\text{15-24}}$	-2.673	0.038	ψ_{15-24}	0.116	0.012
$\alpha^{(\nu)}_{\text{25-44}}$	3.878	0.177	$\alpha^{(\phi)}_{\text{25-44}}$	-2.371	0.026	ψ_{25-44}	0.059	0.006
$\alpha^{(\nu)}_{\text{45-65}}$	2.750	0.230	$\alpha^{(\phi)}_{\text{45-65}}$	-2.392	0.023	ψ_{45-65}	0.051	0.005
$\alpha^{(\nu)}_{\text{66-79}}$	2.302	0.259	$\alpha^{(\phi)}_{\text{66-79}}$	-2.134	0.028	ψ_{66-79}	0.079	0.010
			$\alpha^{(\phi)}_{\text{80+}}$	-1.195	0.019	ψ_{80+}	0.049	0.008
			$\beta^{(\phi)}_{\text{day of the week Tuesday}}$	0.313	0.021			
			$\beta^{(\phi)}_{\text{day of the week Wednesday}}$	0.067	0.022			
			$\beta^{(\phi)}_{\text{day of the week Thursday}}$	-0.053	0.022			
			$\beta^{(\phi)}_{\text{day of the week Friday}}$	-0.024	0.022			
			$\beta^{(\phi)}_{\text{day of the week Saturday}}$	-0.405	0.023			
			$\beta^{(\phi)}_{\text{day of the week Sunday}}$	-0.629	0.024			
$\beta^{(\nu)}_{\text{weekend}}$	-0.863	0.090						
$\beta^{(\nu)}_{\text{public holiday}}$	-0.748	0.411	$\beta^{(\phi)}_{\text{public holiday}}$	-0.208	0.062			
$\gamma^{(\nu)}$	1.927	0.185	$\gamma^{(\phi)}$	0.557	0.019			
$\delta^{(\nu)}$	-2.233	0.027	$\delta^{(\phi)}$	1.849	0.011			
						$\log\kappa$	-0.247	

would expect. Compared with the original model in the main manuscript there is now a greater effect of $\beta^{(\nu)}_{\text{day of the week Friday}}$.

3.2 Option 2: Weight shift from school to household during school holidays

The second option adjusts the household weight based on the reduction of the school weight through the holiday score. The reasoning behind this is that, contrary to the approach in Option 1, the contribution of household contacts expected to increase during school holidays. This means that the school weight decreases with the holiday score (as can be seen in Equation (4)) and the difference from the original school weight to the holiday score-adjusted is added to the household weight. The corresponding equation is:

$$d_{s,t}|_{\text{household}} = d_{s,t}|_{\text{household}} + c \cdot (d_{s,t}|_{\text{school}} - d_{s,t}|_{\text{school}} \cdot h_{s,t}) \qquad (5)$$

where c denotes the fraction of reduced weight in the school setting that is ad-

ded to the household setting. For this analysis we set $c = 0.5$ as we assume that the number of contacts is lower when individuals stay at home instead of going to school. The effect is shown in Figure S7.

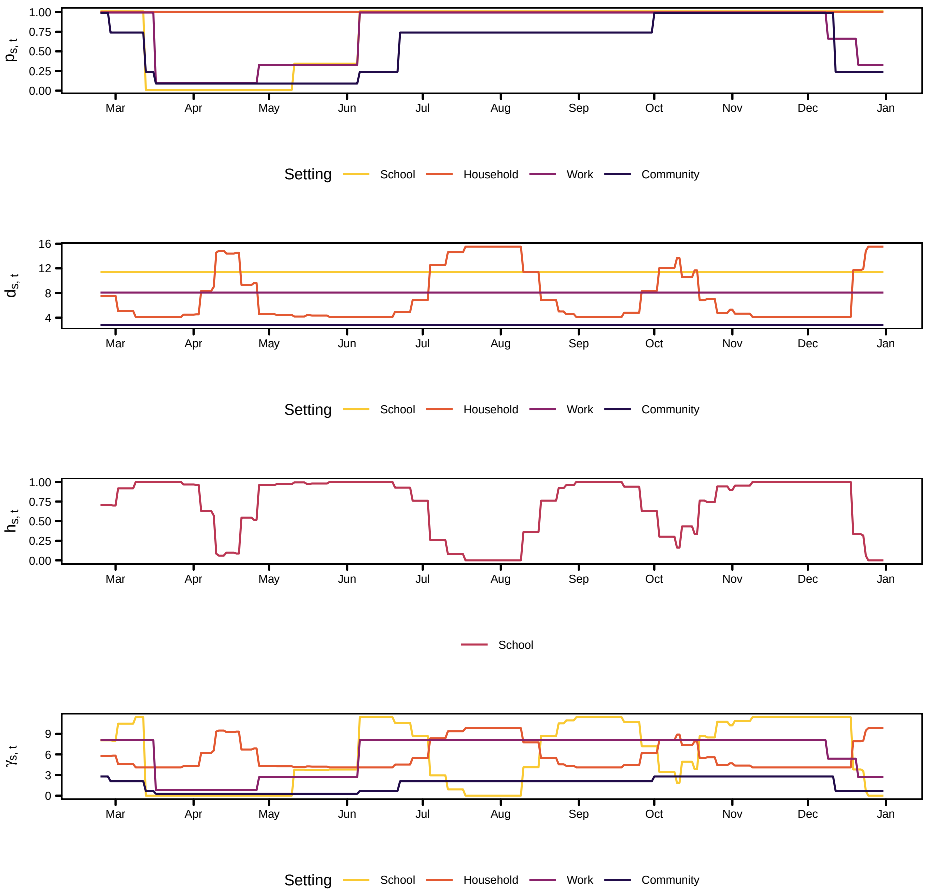

Figure S7: Time series of policy indicators $p_{s,t}$, disease weights d_s, and non-pandemic school holiday score $h_{s,t}$. The lower panel shows the product of all of the time series $\gamma_{s,t}$ which will serve as a multiplication factor for the setting specific contact matrix $c_{a,a',t}$

The corresponding plot and table for Option 2 can be found in Figure S8 and Table S4.

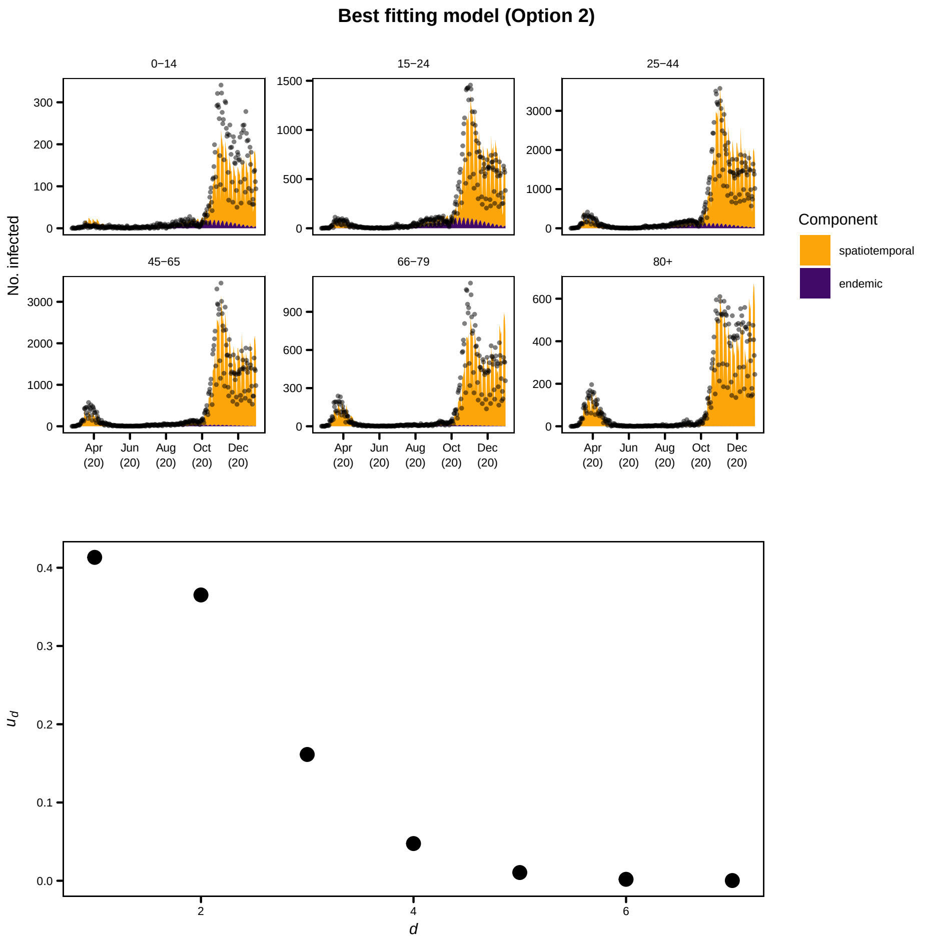

Figure S8: Model fit and lag distribution for the best model with transmission weights $w_{a,a',t}$ as constructed under Option 1. The endemic mean is fitted with effects for age, public holidays, weekday, and sine-cosine wave whereas the epidemic mean is fitted with effects for age, public holidays, weekday, and sine-cosine wave

The model fit shown in Figure S8 is similar to that seen for Option 1. However, the reduction in cases in November and December is not captured as well. Once again, the peak of the estimated lag distribution is at 1. Now the model contains weekday effects in the endemic component (Table S4). Thursday seems a particularly important day for case reporting as it has opposite effects in the two components. The overdispersion parameter ν takes low values but still shows some

Table S4: Coefficients of the best model using BIC under Option 2 and Scenario A

Endemic – $\log(\nu_{at})$			Epidemic – $\log(\phi_{at})$			Other parameters		
Coefficient	Estimate	Std. Error	Coefficient	Estimate	Std. Error	Coefficient	Estimate	Std. Error
$\alpha^{(\nu)}_{\text{0-14}}$	2.520	0.197	$\alpha^{(\phi)}_{\text{0-14}}$	-3.950	0.057	ψ_{0-14}	0.231	0.031
$\alpha^{(\nu)}_{\text{15-24}}$	4.574	0.152	$\alpha^{(\phi)}_{\text{15-24}}$	-2.619	0.036	ψ_{15-24}	0.090	0.010
$\alpha^{(\nu)}_{\text{25-44}}$	3.797	0.156	$\alpha^{(\phi)}_{\text{25-44}}$	-2.302	0.024	ψ_{25-44}	0.054	0.006
$\alpha^{(\nu)}_{\text{45-65}}$	2.512	0.250	$\alpha^{(\phi)}_{\text{45-65}}$	-2.315	0.023	ψ_{45-65}	0.062	0.006
$\alpha^{(\nu)}_{\text{66-79}}$	2.017	0.301	$\alpha^{(\phi)}_{\text{66-79}}$	-2.058	0.029	ψ_{66-79}	0.099	0.012
			$\alpha^{(\phi)}_{\text{80+}}$	-1.129	0.022	ψ_{80+}	0.080	0.013
$\beta^{(\nu)}_{\text{day of the week Tuesday}}$	0.128	0.099	$\beta^{(\phi)}_{\text{day of the week Tuesday}}$	0.355	0.025			
$\beta^{(\nu)}_{\text{day of the week Wednesday}}$	0.362	0.087	$\beta^{(\phi)}_{\text{day of the week Wednesday}}$	0.074	0.025			
$\beta^{(\nu)}_{\text{day of the week Thursday}}$	0.410	0.084	$\beta^{(\phi)}_{\text{day of the week Thursday}}$	-0.066	0.025			
$\beta^{(\nu)}_{\text{day of the week Friday}}$	0.255	0.094	$\beta^{(\phi)}_{\text{day of the week Friday}}$	0.034	0.026			
$\beta^{(\nu)}_{\text{day of the week Saturday}}$	-0.322	0.103	$\beta^{(\phi)}_{\text{day of the week Saturday}}$	-0.454	0.026			
$\beta^{(\nu)}_{\text{day of the week Sunday}}$	-0.791	0.111	$\beta^{(\phi)}_{\text{day of the week Sunday}}$	-0.665	0.026			
$\beta^{(\nu)}_{\text{public holiday}}$	-0.167	0.316	$\beta^{(\phi)}_{\text{public holiday}}$	-0.706	0.071			
$\gamma^{(\nu)}$	1.994	0.160	$\gamma^{(\phi)}$	0.683	0.018			
$\delta^{(\nu)}$	-2.481	0.026	$\delta^{(\phi)}$	1.515	0.013			
						$\log \kappa$	-0.124	

age-dependence. The fixed effects of age group $\alpha^{(\phi)}$ are once again showing a decreasing pattern.

3.3 Discussion

Our goodness-of-fit criterion for model selection–Bayesian information criterion (see previous section)– selects the same model as the main manuscript for Option 1 but a different model is selected for Option 2. This implies our results are robust to the shift in household contacts reflecting policy but not for that reflecting school holiday. Weekly fluctuations remain an important effect to capture in our models for daily case counts.

4 Sensitivity analysis of contact matrices – final size estimates

Considering the options outlined in the previous section, we examine the effect these changes to $\gamma_{s,t}$ has on our predicted case counts. The previous section considered only those results which are relevant for the model selection procedure, i.e. the model fit under scenario A and goodness-of-fit. Now we evaluate the predictions under the two alternative scenarios B and C. Recall, the two options for

sensitivity analysis are:

Option 1: The household weight $d_s|_{\text{household}}$ reflects the school closure policy and is given by $d_s|_{\text{household}} = 2 - p_{s,t}|_{\text{school}}$ ("Household contacts depend on school closures")

Option 2: The household weight $d_s|_{\text{household}}$ is adjusted by any amount which reduces contacts on school holidays (the adjustment is now through $h_{s,t}$) ("Weight shift from school to household during school holidays")

4.1 Scenario B

We now present the effect these changes have on the ratios of the predicted number of counts between Scenario A and Scenario B (Figure S9). We also compare the final size between scenarios A and B in order to simulate the number of cases when schools would have stayed open.

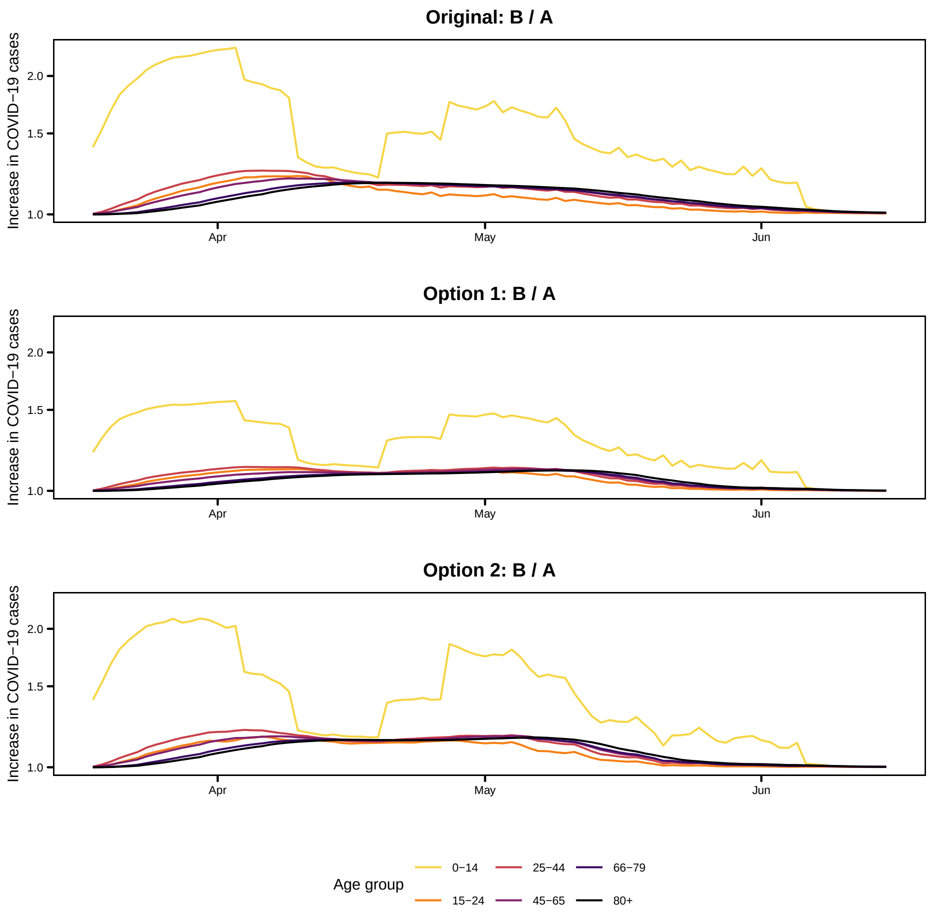

Figure S9: Ratio of the age group-specific path trajectories (B divided by A) throughout time

Both options dampen the number of cases as would be expected since the dominance of contacts in school settings (represented through $d_{s,t}$ is now lower. Option 1 has a lower number of expected cases than Option 2. Both have similar trickle effects to other age groups but the peak is lower for the school and working aged age groups (all ages up to 65) and later for the oldest age groups (ages 66 and above).

Table S5: Comparison between scenarios A (true measures) and B (schools open) for the original method

Age	B - A			B / A		
	P_{10}	P_{50}	P_{90}	P_{10}	P_{50}	P_{90}
0-14	162.9	240.0	404	1.76	1.81	1.87
15-24	45.1	89.9	218	1.06	1.08	1.12
25-44	192.9	362.3	821	1.10	1.12	1.15
45-65	153.3	311.5	773	1.07	1.09	1.12
66-79	48.9	113.8	323	1.04	1.06	1.08
80+	37.3	90.5	271	1.04	1.05	1.07
Total (summed)	641.7	1,207.1	2,820	1.09	1.11	1.13

Table S6: Comparison between scenarios A (true measures) and B (schools open) for Option 1

Age	B - A			B / A		
	P_{10}	P_{50}	P_{90}	P_{10}	P_{50}	P_{90}
0-14	114.3	229	701	1.31	1.36	1.40
15-24	45.4	137	605	1.04	1.06	1.07
25-44	171.0	477	2,014	1.06	1.07	1.08
45-65	155.6	497	2,334	1.04	1.05	1.06
66-79	64.5	248	1,301	1.03	1.04	1.05
80+	53.9	224	1,236	1.02	1.03	1.05
Total (summed)	604.9	1,815	8,188	1.05	1.06	1.07

Table S7: Comparison between scenarios A (true measures) and B (schools open) for Option 2

Age	B - A			B / A		
	P_{10}	P_{50}	P_{90}	P_{10}	P_{50}	P_{90}
0-14	79.4	117.5	232	1.49	1.51	1.53
15-24	24.4	58.3	181	1.04	1.06	1.11
25-44	101.4	223.1	662	1.06	1.09	1.13
45-65	89.7	223.2	719	1.05	1.08	1.11
66-79	36.8	109.6	387	1.04	1.07	1.10
80+	29.0	90.6	328	1.04	1.07	1.10
Total (summed)	359.8	823.6	2,517	1.06	1.09	1.12

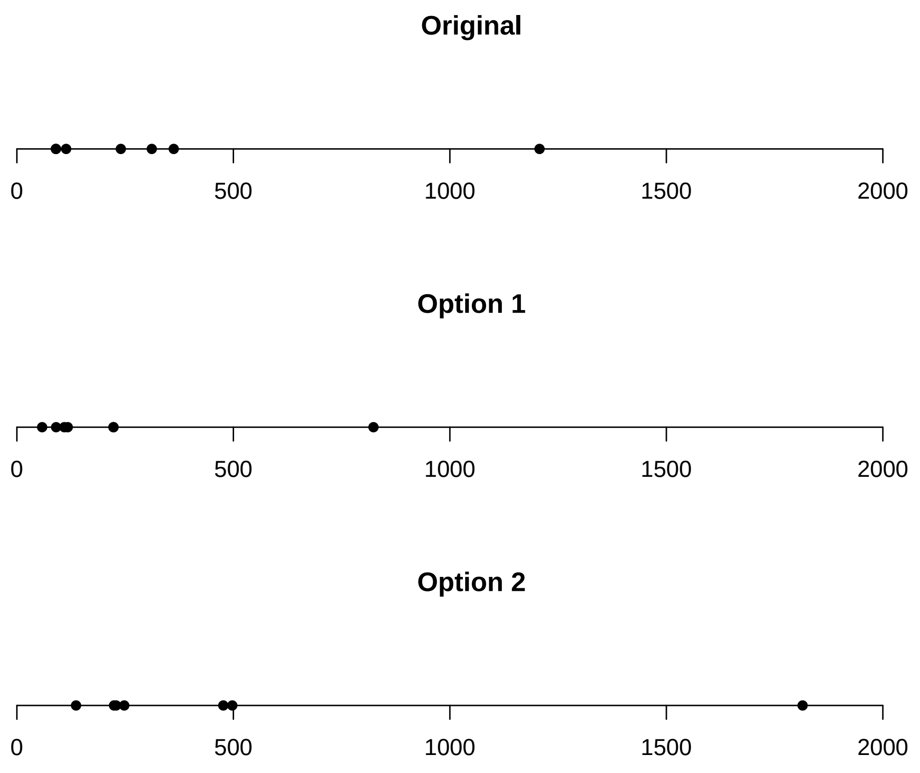

Figure S10: Visual comparison of the P_{50} values for the three options

No difference is found in the ratios for scenario B so the relative measures are the same in this instance. The case counts are greater for Option 2 and lower for Option 1 (Figure S12).

4.2 Scenario C

We also examine the predicted final of the epidemic when schools stay closed under the two alternative options for transmission weights. Now the difference in path trajectories is not as obvious, which means the differences seen for scenario B could also be an artefact of fewer cases early in the pandemic. The decrease in cases for June and July seems larger for the unadjusted weights which again is due to the dominance of contacts in school settings. Here the difference between Option 1 and Option 2 is still apparent with Option 1 having a smaller decrease and Option 2 showing a greater decrease.

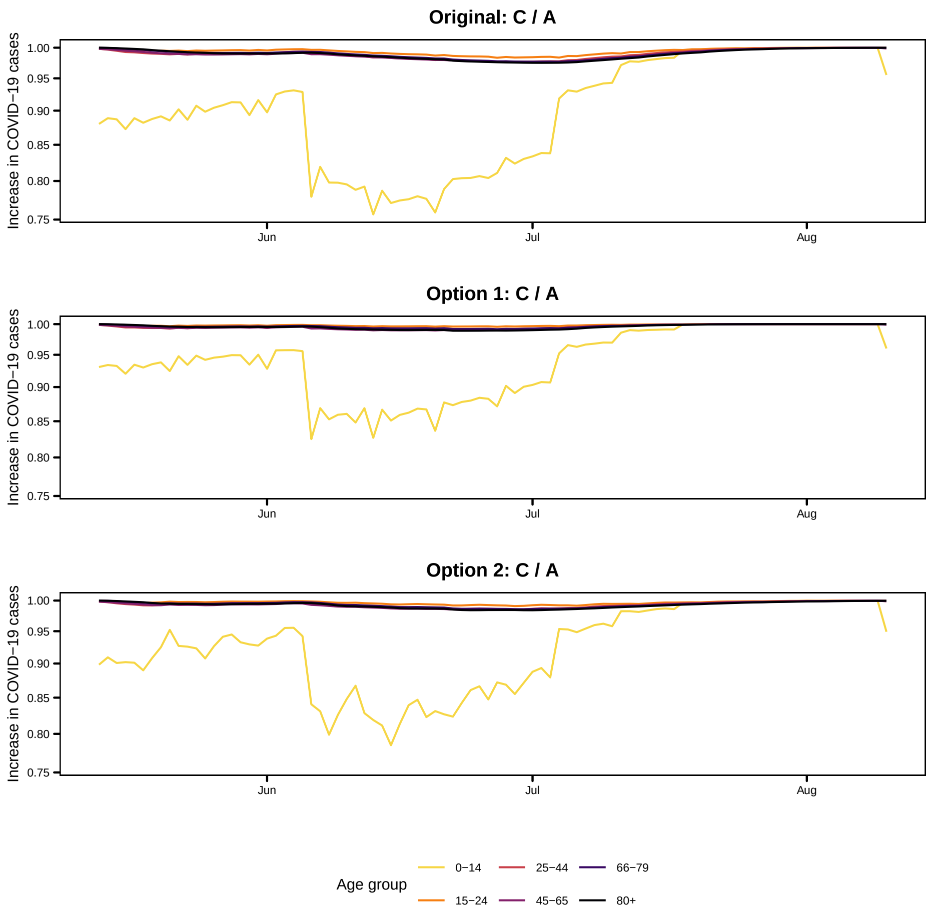

Figure S11: Ratio of the age group-specific trajectories (C divided by A) throughout time

4.3 Discussion

The comparison of scenarios A and B as well as A and C for the various options showed a lot of differences for the adjusted versions against the original. The youngest age group remains the most visible in the path trajectories and the spread of

Table S8: Comparison between scenarios A (true measures) and C (schools closed) for the original method

Age	C - A			C / A		
	P_{10}	P_{50}	P_{90}	P_{10}	P_{50}	P_{90}
0-14	-27.5	-20.85	-16.98	0.915	0.922	0.928
15-24	-16.6	-9.38	-5.98	0.990	0.994	0.995
25-44	-64.4	-36.03	-23.15	0.985	0.989	0.991
45-65	-55.2	-30.08	-18.96	0.983	0.988	0.990
66-79	-13.5	-6.92	-4.05	0.984	0.988	0.991
80+	-9.5	-4.69	-2.62	0.982	0.987	0.989
Total (summed)	-186.0	-107.83	-71.69	0.983	0.987	0.990

Table S9: Comparison between scenarios A (true measures) and C (schools closed) for Option 1

Age	C - A			C / A		
	P_{10}	P_{50}	P_{90}	P_{10}	P_{50}	P_{90}
0-14	-14.29	-11.81	-10.19	0.957	0.959	0.961
15-24	-8.81	-5.16	-3.48	0.995	0.996	0.997
25-44	-35.16	-20.76	-14.12	0.992	0.994	0.995
45-65	-31.28	-17.76	-11.73	0.991	0.993	0.994
66-79	-9.24	-4.83	-2.93	0.991	0.994	0.995
80+	-6.20	-3.17	-1.82	0.991	0.993	0.994
Total (summed)	-104.77	-63.34	-44.47	0.991	0.993	0.994

Table S10: Comparison between scenarios A (true measures) and C (schools closed) for Option 2

Age	C - A			C / A		
	P_{10}	P_{50}	P_{90}	P_{10}	P_{50}	P_{90}
0-14	-8.889	-8.124	-7.522	0.963	0.965	0.966
15-24	-1.589	-1.244	-1.009	0.999	0.999	0.999
25-44	-6.902	-5.590	-4.700	0.997	0.998	0.998
45-65	-5.499	-4.409	-3.678	0.997	0.997	0.997
66-79	-1.036	-0.758	-0.580	0.998	0.998	0.998
80+	-0.691	-0.493	-0.359	0.997	0.997	0.998
Total (summed)	-24.524	-20.604	-17.903	0.996	0.997	0.997

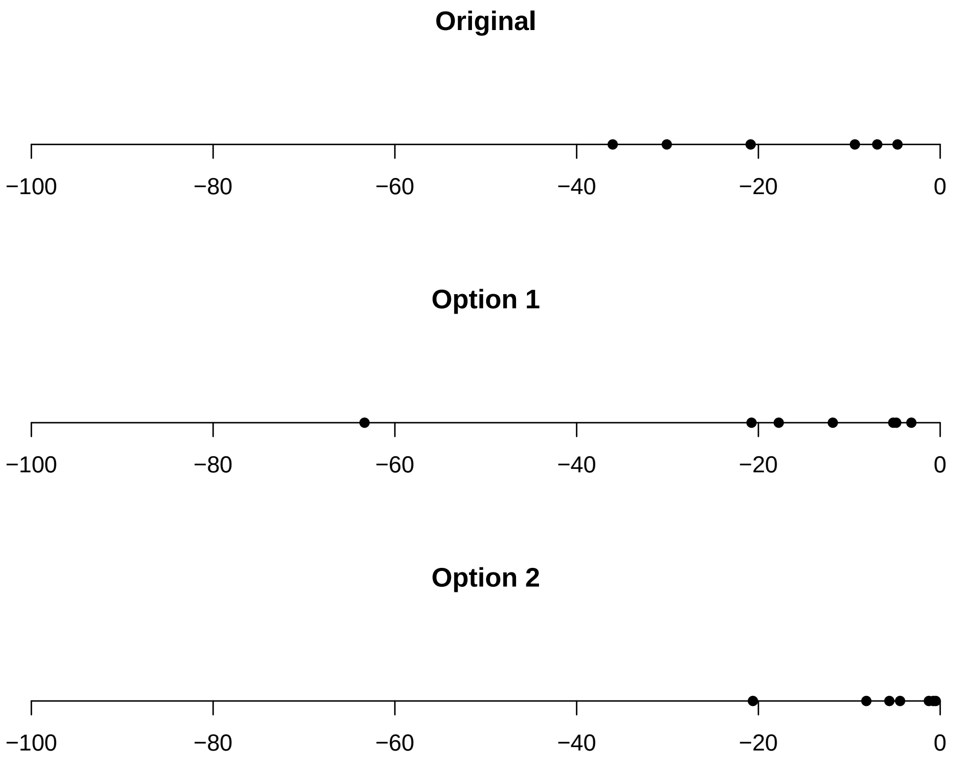

Figure S12: Visual comparison of the P_{50} values for the three options

cases to other ages groups is more evident early on when schools are open (scenario B) than the reduction in cases later on when schools are closed (scenario C).

References
